# Hybrid PolyLingual Object Model: An Efficient and Seamless Integration of Java and Native Components on the Dalvik Virtual Machine

**DOI:** 10.1155/2014/785434

**Published:** 2014-06-12

**Authors:** Yukun Huang, Rong Chen, Jingbo Wei, Xilong Pei, Jing Cao, Prem Prakash Jayaraman, Rajiv Ranjan

**Affiliations:** ^1^Tongji University, Shanghai 200092, China; ^2^Jiangxi University of Finance and Economics, Nanchang 330029, China; ^3^Shanghai Kortide Century Technology, Shanghai 201203, China; ^4^Academy of Space Technology, Nanchang University, Nanchang 330031, China; ^5^CSIRO, Canberra, ACT 2601, Australia

## Abstract

JNI in the Android platform is often observed with low efficiency and high coding complexity. Although many researchers have investigated the JNI mechanism, few of them solve the efficiency and the complexity problems of JNI in the Android platform simultaneously. In this paper, a hybrid polylingual object (HPO) model is proposed to allow a CAR object being accessed as a Java object and as vice in the Dalvik virtual machine. It is an acceptable substitute for JNI to reuse the CAR-compliant components in Android applications in a seamless and efficient way. The metadata injection mechanism is designed to support the automatic mapping and reflection between CAR objects and Java objects. A prototype virtual machine, called HPO-Dalvik, is implemented by extending the Dalvik virtual machine to support the HPO model. Lifespan management, garbage collection, and data type transformation of HPO objects are also handled in the HPO-Dalvik virtual machine automatically. The experimental result shows that the HPO model outweighs the standard JNI in lower overhead on native side, better executing performance with no JNI bridging code being demanded.

## 1. Introduction

In recent years the Android system has become one of the mainstream mobile operating systems that support applications written in Java. As a core technology in Java, Java Native Interface (JNI) is widely used to call the native code from Java and as vice [1]. It is also applied in integrating and reusing the third-party components in Java programs for better performance or to take advantages of hardware features [2–4]. However, despite the claim in [5] that using the JNI with the native code is faster than using Java virtual machine, the communication delay in JNI is not negligible and significant overhead in JNI is still left unsolved. To make it worse, the coding complexity of JNI always makes the developing process inconvenient and cumbersome. Operating efficiency and programming complexity of JNI are more salient on the Android platform, owing to insufficient hardware resources and power limitation as well as frequently visiting peripheral devices. Therefore an efficient and seamless method is needed to reuse native components in an Android application.

In the last two decades, a great deal of research has been dedicated to the improvement of JNI mechanism either for better efficiency or for lower coding complexity. On one hand, efficiency was improved through speeding up the invoking process of native methods with optimized just-in-time (JIT) compiler [6, 7] or by reducing the cost of symbolic lookups for the Java fields and objects accessing [8]. On the other hand, using native code always increases the complexity of application development. To avoid the low level and complex JNI code, solutions such as share stub, bridge liberally or middleware [9–19], automated bridge code, or interface generator [20–22] were introduced. Some of the efforts mentioned above could be adopted into the Dalvik virtual machine (VM) of the Android platform. However, efforts to improve the efficiency of JNI follow the JNI paradigm which is too complicated to be easily used, while efforts to reduce the complexity of JNI tend to lower down the running efficiency.

One of the reasons that JNI cannot be substituted is that most of the native objects have no metadata and reflection ability, so they are incapable of being recognized by the Java VM and be accessed or managed directly by the Java VM. In this case, data have to be transferred between different executions spaces through the JNI functions, which have been proved to be inconvenient and uneconomic.

To solve this problem for the Android platform, we propose a hybrid polylingual object (HPO) model to merge the native component objects into the Dalvik runtime based on the component assembly runtime (CAR) technology [23]. CAR supports the metadata and reflection mechanism for the native components at runtime. We design a metadata injection mechanism to inject the metadata of the CAR component object into the metaclass of a Java object. Under the mechanism, a CAR object can be managed and accessed directly as a Java object and as vice by the Dalvik VM, which greatly improves the execution efficiency of JNI applications. Contributing to the avoided bridging code, the HPO model lowers down the coding complexity substantially.

The rest of this paper is organized as follows. In Section 2, JNI related work is introduced for the improvements of efficiency and usability (complexity).  Section 3 gives an outline of the motivation of the HPO model. In Section 4, details and features of the HPO model are proposed. A modified Dalvik VM for the HPO is designed in Section 5 to support the metadata injection.  Section 6 provides an experiment to testify our approach and the performance. The last section gives the conclusion and discusses the further study.

## 2. Related Work

### 2.1. Research on JNI Efficiency

Besides the help of visiting hardware resources via native code, JNI enhances the efficiency of Java programs by means of the fast execution of native code, as proved in the studies of [24–26]. For example,Batyuk et al. [24] benchmarked the performance of the Dalvik Java code and the native code on Android devices and found that native C applications can be up to 30 times as fast as an identical algorithm running in Dalvik VM, and Java applications can become a speedup of up to 10 times if utilizing JNI.

However, overheads of JNI functions are disturbing which are often observed to pull down the running performance of native code. In order to improve the invoking efficiency of JNI functions, Stepanian et al. [6] introduced a strategy for inline native functions into Java programs using a JIT compiler. This strategy can substantially reduce the overhead of performing JNI calls. They made further optimizations to transform inline call backs into semantically equivalent lightweight operations. Yu-Hsin et al. [7] modified the Java Native Access (JNA) source code and integrated the LLVM JIT compiler into JNA to improve the performance. Lee et al. [8] found that symbolic lookups of accessing Java fields or objects are expensive and can be avoided by caching field IDs and method IDs in static variables in native programs. Yann-Hang proposed to pin objects to their current memory location to ensure that the addresses can be used in future. The Jaguar project in [27] helped Java applications to efficiently access system resources through compile time translation of certain Java bytecodes to inline machine code segments, and a preserialized objects mechanism is advised to reduce the cost of Java object serialization.

### 2.2. Research on JNI Complexity

Using native code with JNI always increases the programming complexity. As Bubak et al. [28] had claimed, the functionality of JNI is available through a complex set of routines and many low level details involving field IDs, method IDs, and class references, which makes the development process long, inconvenient, and error prone. Furthermore, in order to reuse a third-party component in a Java application, a wrapper component or bridge code that delegates to the third-party component is needed [29, 30]. However, native language programmers have to pay extra effort to attach or detach various Java objects explicitly from native code to access Java object's fields or to avoid resource leak [31].

Lots of research aimed at hiding the JNI layer from developers. To reduce the coding complexity of JNI and to help developers write less JNI wrapper code, several tools, including JNA, Simplified Wrapper and Interface Generator (SWIG) [21], Java-C automatic wrapper (JACAW) [32], and AWGNL [33], have been developed to make the bridging process automatic and simple. Wrapper generator shields the user from the details of JNI. For example, SWIG processes an interface file that defines all the variables and functions that need to be accessed from Java and generates the JNI interface to the C code for Java. However, the convenience of these tools comes at the cost of a more complex and cumbersome bridge interface which produces more additional overhead.

In addition to the method of automatic wrapper code generator, several researches hide the JNI layer from developers in other solutions. The Janet package described in [28] provides a language extension which allows incorporating native code directly into Java source files. Jeannie designed in [34] proposed a new foreign functional interface that makes programmers write both the Java code and the native code in the same file. Gabrilovich and Finkelstein [22] suggest a template-based framework that provides automatic selection of the right functions to access Java objects based on their types.

For Windows-specific solutions, many projects, such as Jawin [12], RJCB, Bridge2Java [35], Microsoft VM [36], and Jacob [37], have emerged in the early years that enable interactions between Java and COM components. For example, Jacob uses the bridge technique to bind Java objects and COM objects. Bridge2Java uses a proxy generation tool to parse typelib file and transform the dispatch interface of COM server objects to objects and methods of Java agents. Microsoft Java virtual machine integrates two runtime environments—Java and COM, and each type of objects are packaged individually.

Although these technologies reduce the difficulty of the JNI development, they usually introduce substantial overhead in native function calls. Furthermore, most of the mentioned technologies have application constraints and platform dependency, which makes them hard to be applied in the development of JNI application on the Android platform. For Android-platform solutions, the Android NDK is helpful to reuse a large mass of legacy C/C++ code in Android application, and SWIG is also used to create the bridge code or wrapper code for the native components in Android application [38, 39]. Unfortunately, none of them resolved the overheads produced by the JNI function calls.

## 3. Analysis the Deficiency of JNI

This paper analyses the deficiency of the JNI mechanism at two points, accessing data and invoking methods. At the point of accessing data, critical overhead occurs during invoking native functions from Java to native and even larger ones during invoking back from native to Java to access the Java methods and data. This is because the JNI mechanism can be regarded as a policy that part of the computing ability of a Java object is migrated to the native side while the fields and data structures are still in the Java side. Then native code has to access these resources via the JNI functions, which leads to significant overhead. As a conclusion, the more frequently a Java object is accessed and operated by a native method, the less efficiently a JNI program performs. At the point of invoking methods, JNI links a native function to a Java native method one by one such that the native code is tightly coupled with the Java code and hard to be updated dynamically. Furthermore, the native functions are grouped in libraries in a flat organizational structure, which increases the cost of the symbolic lookups.

As a solution, if all the Java object's fields that are frequently accessed by the native code were moved to the native side, the overheads of the JNI function calls will be greatly reduced. On the other hand, functions in an object-oriented language must belong to a particular class and be invoked upon the class or an instance of the class. So the native method of a Java class can be mapped to a method that belongs to a native class instead of being mapped to an individual function in native libraries. Following these concepts, both the computing abilities and the data resources of a Java class can be encapsulated into a native class. Similarly, a native class can map its computing abilities and data resources to a Java class too. Instead of using the JNI bridging code to integrate and interoperate between these two kinds of heterogeneous objects, we tend to couple them into a hybrid polylingual object (HPO). The HPO model has the ability of directly interacting with both the Java code and the native code on our modified Dalvik VM. Higher executing and developing efficiency of the HPO model is expected because of the avoided JNI bridging code.

The key point of our proposition is to couple a Java object and a native object together in a virtual machine, which will be achieved through the metadata and reflection mechanism of the CAR technology [40]. Java is distinguishable from C/C++ in its reflecting property and metadata maintained. Java objects always carry their own type information with them. The Java's metaprogramming is based on its core reflection API, which allows inspection of types and construction of dynamic method calls [41, 42]. However, C/C++ has no metadata and reflection mechanism. Although a COM object supports metadata, it obtains limited reflection ability through the IDispatch interface and automation techniques [36, 43], which makes it not a good choice for Android applications. The CAR technology is finally chosen to develop native components on the Android platform because of the following. (1) The CAR technology is qualified to be used in an embedded system. (2) CAR has a programming-language independent component standard, and a CAR component can be implemented in C/C++ or other compiled languages. (3) CAR supports metadata and reflection mechanism.

Contributions of this paper are the following. Firstly, we present a HPO model allowing native component object to be accessed directly as a Java object and vice versa. It helps to reduce the overheads of JNI function calls. Secondly, in order to support the HPO model in the Dalvik VM, we propose a metadata injection approach that provides automatic mapping and reflection ability to combine the Java object with the native object. Thirdly, we provide the HPO-oriented development tools that help developers to implement the integration and conversion between the Java components and the native components on the Android platform. With the help of these tools, developers no longer need to write the bridging code for the heterogeneous components or to struggle with the details of JNI programming.

## 4. Hybrid Polylingual Object Model

In this section, a hybrid polylingual object (HPO) model is proposed to achieve the integration and interoperation between Java components and native CAR (C++) components. The definition of the HPO model is given, followed by the features being discussed.

### 4.1. Definition

**Figure 1:**
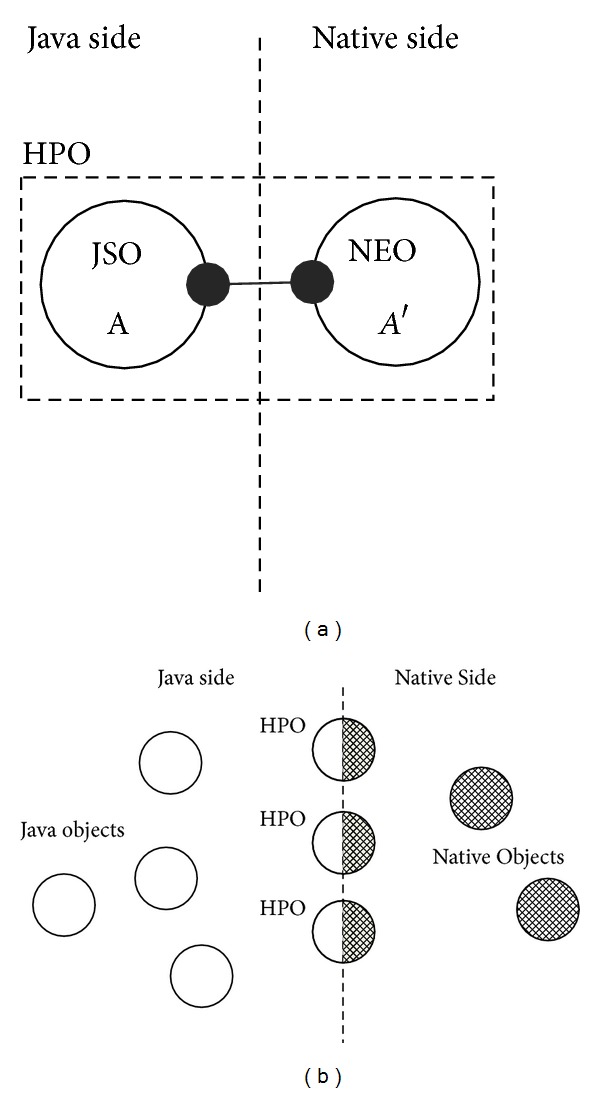
Hybrid polylingual object model and runtime environments. (a) A single HPO. (b) A runtime environment supporting the objects for Java, HPO, and CAR component.

In general, a HPO has some interoperation interfaces for both Java and CAR programs. The object is defined as a Java class and acts as a normal Java object in the VM, while it is implemented by the native object to improve the running efficiency. A schematic plot of the proposed HPO model is shown in Figure 1(a), in which a hybrid polylingual object is composed of a Java object and a CAR object which are bound through the Dalvik VM. The Java object is named as Java stub object (JSO) and the native object is native entity object (NEO). Based on the HPO model, we provide a runtime environment supporting the life of the objects for Java, HPO, and CAR component simultaneously, as shown in Figure 1(b).

**Definition 1 (Java stub object).**

A JSO is a Java object whose status and behaviors are migrated to the native side. It is merely a stub object for the corresponding object existing in the native side.

**Definition 2 (native entity object).**

A NEO is a native object that encapsulates and implements all the status and behaviors of a JSO. It is the real entity that preserves the status and performs the behaviors that belong to a JSO.

**Definition 3 (hybrid polylingual object).**

A HPO is a cross language compound object that is archived by coupling a Java stub object with one or more NEOs together in virtual machine.

The HPO model has the following features. Firstly, though a JSO and a NEO are located in different address spaces, they have same lifespans and live in same threads. Secondly, the language programming paradigms and compiling for JSOs and NEOs are independent. Thirdly, the HPO model is object-oriented because of its encapsulation and inheritance. Lastly, the HPO model is also a component-oriented model, considering the ability of reusing a HPO component through interfaces without source code.

Though HPO has shown potentials of binding Java object with native object and supporting the integration of Java component and CAR components, some constraints have to be followed to get the characteristics mentioned. These constraints are (1) the cross languages inheritance of a HPO model; (2) the mapping relationships and coupling manners between JSOs and NEOs; (3) the injection mechanism of the programming metadata for a HPO class; and (4) The transformation of Java components and CAR components. These constraints will be discussed in detail as follows.

### 4.2. Inheritance in the HPO Model

**Figure 2:**
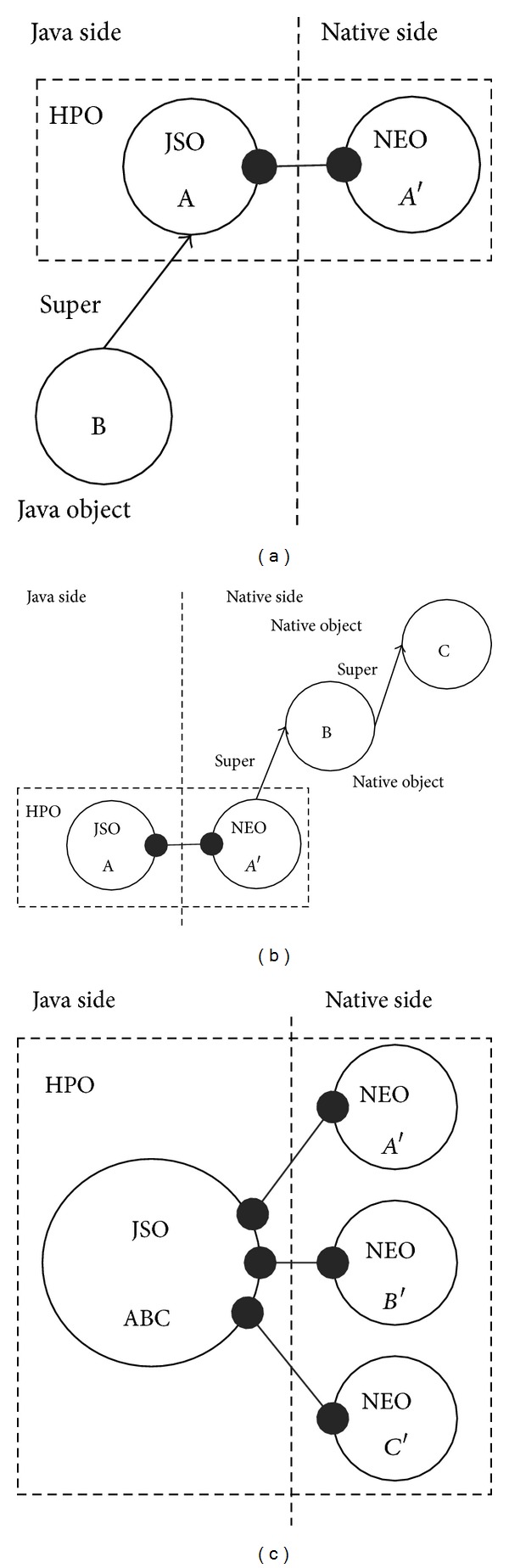
Inheritance of HPO classes. (a) Inheritance in Java side. (b) Inheritance in native side. (c) Interface inheritance.

In the object-oriented programming, inheritance allows a class to pass on its status and behaviours to its children and allows programmers to reuse code. Generally, inheritance can be realized easily in a single program environment, but cross language inheritance of HPO may be more difficult. There are three cases of inheritance for the HPO model, as shown in Figure 2.

In the first case, a Java class is inherited from a HPO class and complies with the Java's inheritance mechanism. In other words, a HPO class derives its children class in a Java environment, as shown in Figure 2(a).

In the second case, a NEO class is inherited from another NEO class iteratively. In this case, all the father or ancestor NEO classes related to the first NEO class could be accessed from the same JSO class that is coupled with the first NEO class, as shown in Figure 2(b).

In the last case, a JSO class may inherit from multiple interfaces, and each interface is implemented by one NEO class. In this case, all the NEO objects should be coupled to the corresponding JSO object, as shown in Figure 2(c).

### 4.3. Mapping and Coupling

**Figure 3:**
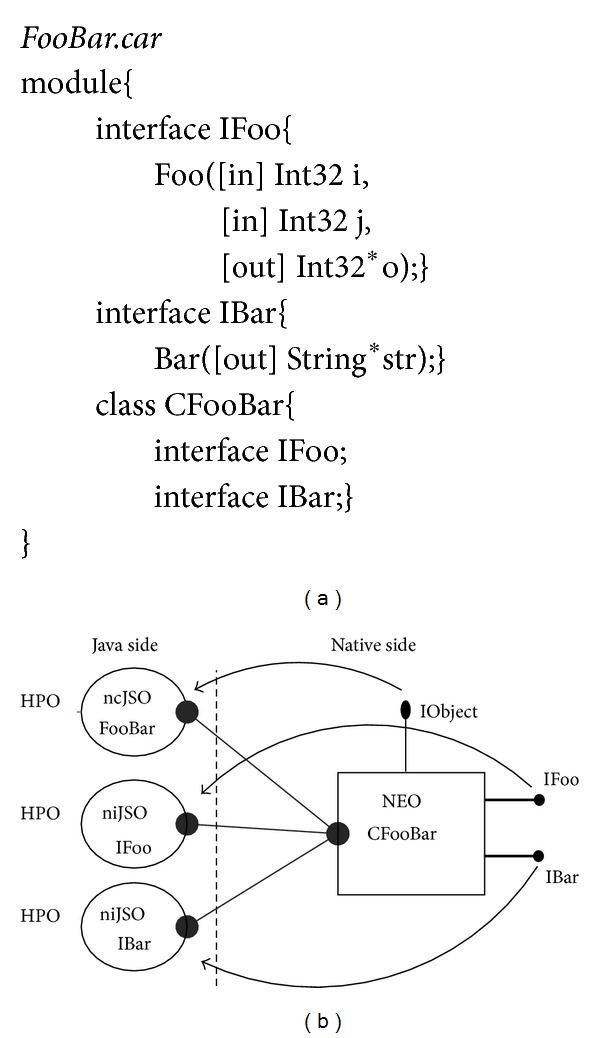
Mapping and Coupling. (a) FooBar component and CFooBar class. (b)The corresponding JSO classes coupled with CFooBar.

In the HPO model, the mapping relationship between the JSOs and the NEOs is not always the one to one. On one hand, one JSO may be coupled with multiple NEOs in the case of multi-interface inheritance. On the other hand, one NEO may be coupled with multiple JSOs because a NEO may have implemented several interfaces and each interface is coupled with a special JSO. For example, Figure 3(a) shows an IDL file of the FooBar component in which the CFooBar class is defined, and Figure 3(b) shows that each interface that belongs to the CFooBar class is mapped to one special JSO.

An IDL file in CAR component technology is a  .car file and is used to describe the interactive contract between Java components and CAR components. CFooBar is a CAR component class defined in the FooBar component and includes two interfaces, IFoo and IBar. In addition to the interfaces defined explicitly in the  .car file, every CAR component class inherits an IObject interface (similar to COM IUnknow) implicitly from CObject. To simplify the mapping relationship of the JSOs and the NEOs, we build a rule on how to map a NEO class to multiple JSOs; each NEO class in a component is mapped to a JSO class, and each interface in a NEO class is mapped to one JSO class, respectively, too.

Following the mapping rule, IObject, IFoo, and IBar are mapped to FooBar.java, IFoo.java, and IBar.java, respectively. However, FooBar.java is different from IFoo.java and IBar.java in two points.

- They are coupled with the CFooBar object through different interfaces. FooBar.java is coupled with the IObject, while IFoo.java and IBar.java are coupled with the IFoo and IBar of the CFooBar, respectively.
- FooBar.java is responsible for instantiating the NEO of the CFooBar class and manages the lifespan, while IFoo.java and IBar.java are exempt from that.

For convenience, the FooBar.java class is marked as a native class JSO (ncJSO), while classes of IFoo.java and IBar.java are marked as native interface JSOs (niJSO).  Algorithm 1 lists the definitions of the JSOs that are mapped from CFooBar.

### 4.4. Metadata and Annotation

In the stage of programming, it is necessary to add the mapping information of the JSOs and NEOs to the Java class source code. The coupling of a JSO and a NEO is implemented by the proposed metadata injection mechanism which is critical to realize (1) how a HPO is defined and used in the programming stage without changing the Java syntax and (2) how a HPO is created and managed in the runtime stage.

The metadata of a HPO class can be divided into two types, the metadata that is used in the programming and compiling stage (programming metadata) and the metadata that is used during the runtime stage (runtime metadata). The runtime metadata is relevant to the VM environment and will be discussed in Section 5.

Programming metadata is composed of the type of a JSO (ncJSO or niJSO), the name of the component module in which the corresponding NEO is defined, and the class name and the interface name of the NEO. These programming metadata of a NEO is injected into the corresponding JSO class source code by means of the annotation mechanism of Java [44]. For each NEO class or interface in a native component, a few annotations are added to its corresponding JSO class. Then a JSO class becomes a HPO class that carries the programming metadata about the NEO. Annotations in Java are a kind of special classes and can be used to comment the classes, methods, variables, parameters, and packages in Java class source code and may be embedded in Java class files by compiler. These annotation classes are recognized by the Java VM at runtime to retrieve values of the embedded elements that are defined in these annotation classes [45]. Therefore, the metadata will be exploited by the Dalvik VM to create instances of the HPO classes. This approach neither has it changed any semantics of the Java class, nor has it changed any syntax of the Java language.

Annotations added to the Java class are described as below.

(a) A Java class is annotated as a ncJSO class by the @HPOClass annotation consisting of two elements: the name of the native component module that defines the NEO class and the name of the NEO class. The @HPOClass annotation is written as
  (1) @HPOClasss(Module=module_name,Class=class_name).
(b) A Java class is annotated as a niJSO class by the @HPOInterface annotation class consisting of two elements: the name of the native component module that defines the NEO interface and the name of the NEO interface. The @HPOInterface annotation is written as
  (2) @HPOInterface(Module=module_name,Interface=interface_name).

It is unnecessary to define any annotations for the native methods in a JSO class source code because the Dalvik VM will automatically mangle the JSO native method with the homonymic method in the corresponding NEO. The definitions of the HPOs converted from CFooBar are shown in Algorithm 2.

### 4.5. Conversion between Java and CAR Components

The HPO model provides a conversion framework between Java components and CAR components in addition to the benefits of reusing legacy code for CAR components and Java components in Android applications. Tools of CAR2Java and Java2CAR are provided to generate the HPO class source code automatically, which reduce the manual participation during the conversion procedure to improve the developing efficiency. CAR2Java and Java2CAR provide the ability of reading IDL files and generating files of HPO classes that consists of  .java (for JSOs),   .h and,  .cpp (for NEOs). These files will be implemented in Java or CAR, respectively, without any JNI specifications being concerned.

Steps to convert an existing Java component to a CAR component are as follows.

- Write an IDL file to define the interfaces between a Java component and a native component, in which each class, interface, or method is extracted from the JSO class in the Java component.
- Annotate all these JSO classes with the @HPOXxx annotation class that is mentioned in Section 4.4.
- Parse the IDL file with Java2CAR to generate the skeleton code (header files and source files) of CAR classes.
- Implement all the CAR classes in the native component.
- Compile the native component to get an.eco (.so) file.
- Put the  .eco (.so) file to the path that is specified by the Java application.
- Build and run the Java application in the Android SDK.

Steps to convert an existing CAR component to a Java component are as follows.

- Write an IDL file to define the interfaces between the CAR component and the Java component, in which each class, interface, or method is extracted from the NEO class in the CAR component.
- Parse the IDL file with CAR2JAVA to generate the source code of the corresponding JSO classes with @HPOXxx annotations inside.
- Write a client Java class to instantiate HPO classes in Java code.
- Put the  .eco (.so) file to the path specified by the Java application.
- Build and run the Java application in the Android SDK.

If neither Java components nor CAR components are provided, the coding can be motivated by writing an IDL file and putting it to the CAR2JAVA/JAVA2CAR tool to generate the skeleton code for both Java and CAR components.

### 4.6. Programming with HPO

Supposed that the FooBar component (as shown in Figure 3) has been implemented in CAR and the corresponding HPO classes have been defined in Java (as shown in Algorithm 2), a normal Java class is required to serve as a client that instantiates the HPO classes; therefor the HPO can be accessed by other Java classes in an Android application. The example code of the client class, FooBarClient.java, is shown in Algorithm 3.

Our approach relieves the Java programmers of learning and using JNI functions. In the Java side, coding in the HPO-based program is almost the same as coding in a JNI-based program; the main difference between these two types lies in the native side. For native code developers, JNI-based programs require the knowledge of JNI functions and JNI specifications. For HPO-based developers, HPO programs require the knowledge of CAR component programing. Since CAR components can be written in C++, a C++ developer can write a CAR component program easily. In a HPO-based program, there is no bridge code between Java and CAR components, so developers write less code and no longer need to struggle with the low level details of JNI.

The HPO model supports dynamic updating of native components in Android applications. A HPO-based Android application consists of two parts: a Java program and one or more native components. These two parts are independent of each other at the compile time; that is, they are dynamically bounded at runtime. Furthermore, these two parts are interacted with each other through an interface. If the interface is kept unchanged, the native components can be dynamically updated and replaced without the need of recompiling the Java code. This is helpful to the migration of HPO programs across platforms.

## 5. Design of the HPO-Dalvik VM

An improved Dalvik VM is implemented in this section to support objects of Java, HPO, and CAR simultaneously, which is achieved by modifying the standard Dalvik VM and embedding the CAR runtime in the Dalvik runtime. The improved Dalvik VM is named as the HPO-Dalvik VM for convenience. A HPO-Dalvik VM addresses the following issues: (1) how to recognize a HPO class and figure out the corresponding JSO class and NEO class; (2) how to load a NEO class and instantiate a NEO; (3) how to couple a JSO and a NEO together and invoke native methods in a HPO dynamically; (4) how to manage the lifespan of a HPO;and(5) how to interconvert data types between Java and CAR. Answers to these issues depend heavily on the proposed mechanism of runtime metadata injection.

### 5.1. Runtime Metadata of HPO

**Figure 4:**
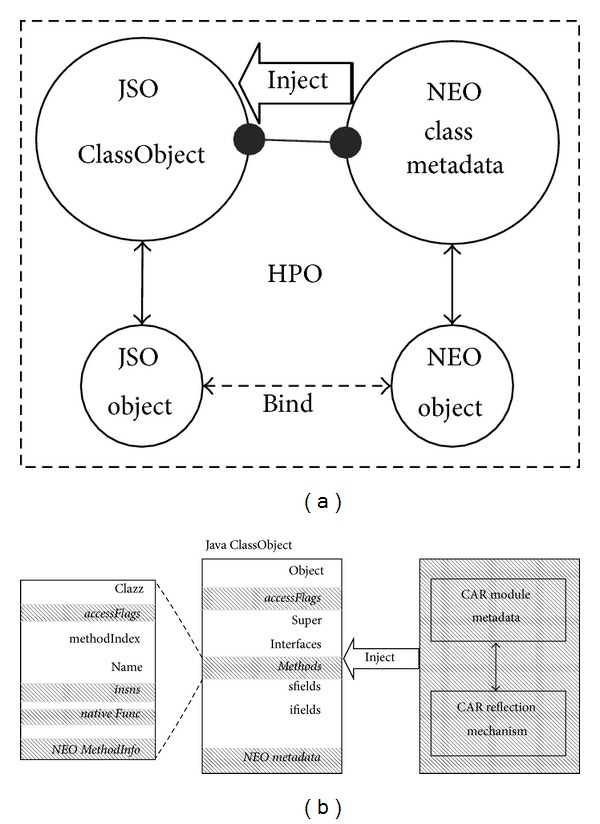
NEO metadata injection. (a) Inject the CAR object metadata into the JSO ClassObject.(b) The modified structure of the JSO ClassObject.

In order to couple a JSO and a NEO at runtime, the NEO runtime metadata is injected into the* ClassObject* of a JSO class. In Java, the metadata of a class is grouped as a sort of metaobject named* ClassObject* and can be used mostly with the reflection. The runtime metadata of a CAR class is a set of descriptive, structural, and administrative data linked to this class and can be retrieved by the CAR reflection of APIs at runtime. These reflection interfaces for CAR object metadata will be injected into the JSO* ClassObject* when a HPO class is loaded at runtime, as shown in Figure 4(a).

Figure 4(b) shows the JSO* ClassObject* structure of the proposed mechanism of runtime metadata injection. By default, all user classes are loaded by the system class loader, but it is possible to replace the default class loader with one or more customized class loaders. Here a new class loader for HPO classes is implemented. Whenever the HPO-Dalvik VM loads a Java class annotated with @HPOClass or @HPOInterface, it sets a flag in the* accessFlags* field of a JSO* ClassObject* to indicate that it is a ncJSO class or a niJSO class. Three* accessFlags* values are added to indicate the type of a HPO class.

- CLASS_CAR_CLASS indicates that the current Java class is a ncJSO class;
- CLASS_CAR_INTERFACE indicates that the current Java class is a niJSO class;
- CLASS_CAR_NEEDCLEAN is used to perform the garbage collection of the NEO object, which indicates that the current JSO class needs to deconstruct its NEO objects.

A HPO-Dalvik VM loads the native component modules and NEO classes of a HPO class by the names of the modules, classes, or interfaces that are specified through the annotations of the HPO class. The HPO-Dalvik VM retrieves the metadata of these NEO classes or interfaces according to the metadata type with the help of the CAR reflection mechanism. The HPO-Dalvik VM injects the references of these metadata into the corresponding fields and structures of the* ClassObject*.

Figure 4(b) also shows the metadata injection of the native methods. In a JNI program, a Java method is specified with the flag “native” to tell the Java VM to search for the method in the native code. Similarly, a native method in a JSO is specified with flag “native” and is bounded to the method that has the same name in a NEO. The exact method to be called is determined at the first invocation at runtime. Before the method is called at the first time, the* nativeFunc* pointer in the* method* structure points to the entry of the HPO-Dalvik dvmResolveNativeMethod() function. This function is modified from its original version in the Dalvik VM to parse the metadata of the corresponding NEO native method and obtain the exact native method address. If the process is successful, the* insns* field in the* method* structure will hold the address of the native method, and the* nativeFunc* field would be assigned with the entry address of the dvmCallCARJNIMethod() function (implemented in HPO-Dalvik to push the arguments of a JSO native method into the native stack and call the CAR native method that is pointed by the* insns* field). A HPO-Dalvik VM does not need to go through the resolving process again unless the HPO is destroyed. And all calls to the JSO's native method are bridged by dvmCallCARJNIMethod() function.

### 5.2. Instantiation of a HPO

The HPO-Dalvik VM treats a HPO as a normal Java object. A HPO can be regarded as an integer object whose lifespan has the same period to a normal Java object: created, in use, invisible, unreachable, collected, finalized, and deallocated. When the* new* operator is performed onto a HPO, the HPO-Dalvik VM analyses the JSO's class file to obtain the value of the attribute CLASS_CAR_TYPE and then determines the CLSID of the corresponding CAR object according to the value of CLASS_CAR_TYPE. During this period, the HPO-Dalvik VM looks up necessary information for creating an instance of the CAR object by the CAR's reflection interfaces.

It is reasonable to take inheritance into account when an instance of a HPO class is created. If no inheritance happens, a HPO object involves only one JSO and one NEO. In this case, the HPO-Dalvik VM must allocate one more unit in the size of u4 (unsigned int) behind the instance object of a JSO to store the reference of a NEO instance object. If inheritance happens, a HPO object may involve one JSO and multiple NEOs. In this case, the HPO-Dalvik VM calculates the number of the NEOs (including the direct NEO as well as its father and ancestors) and allocates corresponding memory in the size of u4 behind the instance object of the JSO to store all the references of these NEO instances. The JSO could access its *n*th NEO on level *n* by calculating the pointer offset according to

(3) $$\begin{array}{c} \text{offset}=\text{objectSize}+\text{sizeof}\left(\text{u}4\right)*n \end{array}$$

### 5.3. Garbage Collection of HPO

The HPO-Dalvik VM manages HPO and Java objects in a unified rule, so the policy of garbage collection for the HPO objects should be the same as that of the normal Java objects. The HPO-Dalvik VM must ensure that all the JSO and NEOs that belong to one HPO have the same lifespan. However, different from the Java garbage collection which manages objects memory implicitly, CAR manages the objects memory by reference counting such as setting the relevant object to null or using release() method to remove the memory explicitly. Our solution is to deconstruct all the NEO objects compulsorily when the JSO object is destroyed by the garbage collectors.

It is worthy of being noted in the case of the movement of Java objects. The Dalvik VM adopts multiple garbage collectors, which cause the movement of a Java object such as copying collectors [46]. Typically, copying collectors are to duplicate Java objects from the old heap to the new heap. Once the duplication is done, the old objects become useless and the reference of the JSO will be changed. Since a NEO keeps the reference of the corresponding JSO at the time of its initialization, it has to update the reference to the new JSO address as soon as a JSO is moved.

### 5.4. Data Types Transformation

Data types mapping between Java and CAR is shown in Table 1. There are two kinds of data types in Java: primitive type and reference type. A primitive type is directly mapped to the counterpart of the CAR. For example, boolean, byte, short, int, long, float, double, and char of Java are mapped to Boolean, Byte, Int16, Int32, Int64, Float, Double and Char of CAR, respectively.

Different from the reference types of Java which are passed as opaque references to native methods in JNI, the HPO-Dalvik VM maps Java reference types to CAR reference types by metadata and reflection. Reference types such as String, StringBuffer, arrays, and Java classes are mapped to the reference types of String, StringBuffer, ArrayOf<Type>, BufferOf<Type>, and CAR classes.

Parameters or objects of each reference type should be converted into a HPO so that they are accessed by a HPO native method. To this end, the corresponding NEO (CAR) objects should already exist in the native side. When the fields and methods of these HPO objects are accessed by a native method, they are actually accessed in its NEO object. The HPO-Dalvik VM will find out the appropriate NEO objects and feed their references to the native method in native side. Take an object type as an example, for a FooBar object in Java, the HPO-Dalvik VM maps it to the type of CFooBar in CAR. If a CFooBar object already exists in the CAR runtime, the HPO-Dalvik VM delivers the CFooBar object reference to the native method that is going to access this FooBar object; otherwise it will create a CFooBar object by CAR's reflection mechanism and deliver the reference of the created object to the native method. Then high performance is expected without the JNI interface method which parses data from the reference parameters or creates and returns a new Java object in the native side.

## 6. Performance Evaluation

In order to validate the feasibility of the presented HPO model, we built a prototype of runtime VM for HPO objects on the basis of the Dalvik VM in Android 2.3.7 and the Elastos component platform. The Elastos component platform is a middleware that provides a runtime environment to support CAR components running on embedded systems [47]. An experimental application on HPO was developed and was compared with the JNI counterpart.

The experiment was carried out on

- CPU: Intel(R) Core (TM) i3-3110 M 2.40 GHz,
- memory size: 2 GB,
- OS: Android2.3.7,
- toolkit: Android NDK R8b,
- device: AVD emulator based on Android2.3.7,
- target: API level 10.

### 6.1. Test Cases and Experimental Method

To evaluate the execution efficiency of calling native methods for HPO and JNI, four types of native methods are prepared:

- public native int Sum(int *n*);
- public native String Strcat(String *a*, String *b*);
- public native int[] ArrayAdd(int[]  *a*, int[]  *b*);
- public native MyObject GetMyObject(MyObject obj).

Native methods were focused on the execution time running in the Java side and in the native side. Logging facility of the Android platform was used to profile the native calls. The system clock was recorded in millisecond units by the function System.currentTimeMillis() to get the CPU time in the Java side and in microsecond units by the function gettimeofday() to get the CPU time in the native side. Each method was repeated for 100, 200, 400, 600, 800, or 1000 times to get a legible figure and an average time.

### 6.2. Experimental Results and Analysis

#### 6.2.1. Calling Native Method from Java Side

Table 2 presents the average execution time of the native methods that are called in the Java side. An outstanding acceleration of the HPO method is clearly observed, though it may vary according to the data types of the method parameters and return value. As an example of simple data types, the average execution time of the HPO-based Sum() method is almost the same as that of the JNI-based Sum() method, with the gap less than 0.5 ms. For the ArrayAdd() and Strcat() methods with complex data type parameters and returning values, the speedup of HPO is quite obvious. For example, under the condition of 400 loops, the JNI-based native method ArrayAdd() costs 30.50 ms, while the corresponding HPO-based method costs only 13.92 ms. Similarly, HPO-based GetMyObject() method of 1000 loops receives a time saving of 69 percent, which indicates that complex data types earn better efficiency improvement than simpler ones.

#### 6.2.2. Running Native Method in Native Side

Table 3 displays the execution time of each test case looped 100, 200, 400, 600, 800, and 1000 times in the native side, and similar results are drawn from Table 2. For simple data types, the execution time of JNI-based Sum() is almost the same as that of HPO-based Sum(). But the execution time of moderate complex data types like HPO-based ArrayAdd() and Strcat() methods reduces to almost half if compared with the corresponding JNI-based methods. For the native method GetMyObject() with complex data types and returning values, the JNI-based implementation spends 1129.12 ms to run 1000 loops in the native side, which is 21 times longer than that of the HPO-based implementation.

#### 6.2.3. Invoking Cost of Native Method

The elapsed time for invoking a native method and returning from it can be obtained by subtracting the average execution time in the native side from the average execution time in Java.

The rightmost two columns in Table 4 show the recorded overhead time for invoking methods and returning process, which show an additional cost of 16% to 114% from the HPO model. The cost of the first three native methods varies from 0.8 *μ*s to 1.4 *μ*s, while the gap is widened to 64 *μ*s in the last method GetMyObject(). This extra cost is caused by the reflection mechanism of the HPO-Dalvik VM reflecting the method metadata and mapping data types of parameters. Owing to the significant speedup of native methods running in native side, the extra invoking cost of the HPO model is acceptable.

The results show that our solution enhances the executing efficiency of calling native methods definitely. There is a performance gain of 10–70% in each call of native methods in a small price for reflection, which indicates that the HPO model is a promising technique for the integration and interoperation of Java components and native components in the Dalvik VM.

#### 6.2.4. The Application Size

From the experiment, HPO-based programs tend to have smaller size, which is meaningful to mobile devices with limited storage space. The HPO program in the test is composed of two parts with total compiled size of 220.6 KB, in which the Java part is 184 KB and the native part is 36.6 KB. However, the total size of the compared JNI program reaches 536 KB. This shrinkage of size can be explained by the avoidable bridging code.

## 7. Conclusions

High complexity and low efficiency of the JNI mechanism are main obstacles that keep developers from integrating and reusing heterogeneous native components in mobile applications, web services, and wireless networks [48–56]. Existing approaches or tools for JNI mechanism fail to solve the efficiency and the complexity of JNI simultaneously, which is even critical in the environment of the Android system.

In this paper, a novel approach to reduce the overhead of the JNI function calls and the complexity of JNI programming is proposed. The solution is to build a HPO model, a cross language compound object that is composed of a Java stub object and one or more native entity objects in a virtual machine. The HPO model helps programmers to operate data and objects in the native side, which escapes from the JNI ways that access through the bridging code. The runtime coupling of a Java object and a CAR object is implemented in a modified Dalvik VM based on the technique of metadata injection. The modified VM provides automatic mapping, allowing any CAR component object to be accessible as a Java object and vice versa. This is a powerful feature that makes all the existing CAR-based applications and services become available to Java. Following the ideas, the HPO-based development tools are presented, and a prototype VM is implemented based on the Dalvik VM and Elastos. Experiments with simple or complex data types demonstrate an initial evidence of the feasibility and effectiveness of the HPO model.

Even though the HPO model has been proved to be useful, some other studies are still on the way to perfection. For example, the storage approach for shared fields needs a deeper insight; the callback mechanism and a cross language debug toolkit are also indispensable to make it prevalent; support to other languages like JavaScript and Python is even difficult. All these challenges lead the direction of our next work.

## Figures and Tables

**Algorithm 1:**
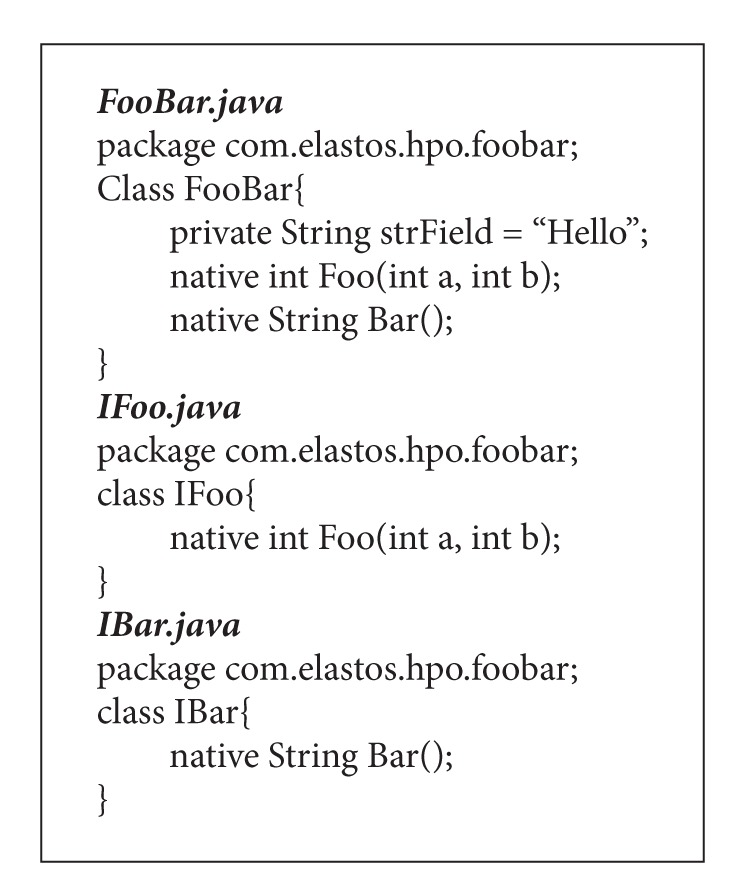
Definitions of JSO classes.

**Algorithm 2:**
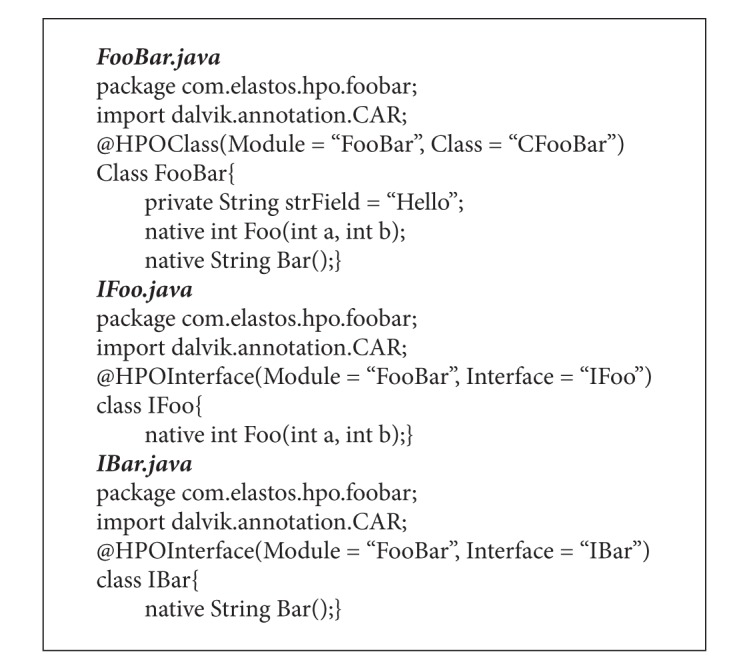
Definitions of HPO classes.

**Algorithm 3:**
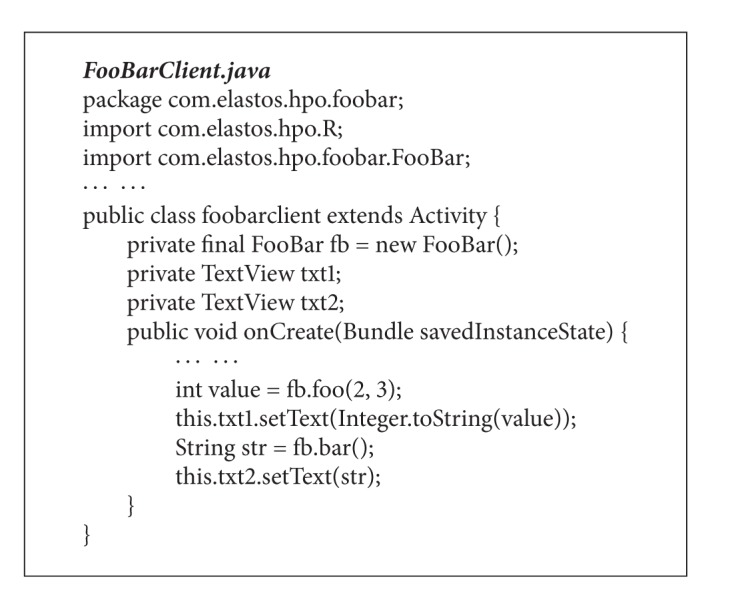
Programming with HPO.

**Table 1:** Data types of Java and CAR.

Java	CAR	Description
boolean	Boolean	Eight bit integer
byte	Byte	Eight bit signed integer
short	Int16	16 bit signed short
int	Int32	32 bit signed integer
long	Int64	64 bit signed long
char	Char16	16 bit unsigned integer
float	Float	32 bit float
double	Double	64 bit float
Object	Interface	object
String	String	String
StringBuffer	StringBuf	String buffer
array []	ArrayOf/BufferOf	array

**Table 2:** Call native method from Java.

From Java (ms)	100		200		400		600		800		1000	
	JNI	HPO	JNI	HPO	JNI	HPO	JNI	HPO	JNI	HPO	JNI	HPO
Sum	0.20	0.21	0.38	0.75	1.00	1.10	1.22	1.24	1.71	1.78	1.80	1.85
ArrayAdd	7.85	0.66	17.00	8.44	30.50	13.92	46.50	28.77	53.30	41.92	86.10	78.60
Strcat	15.12	1.21	23.60	9.06	35.70	28.50	56.43	25.96	95.70	49.12	136.26	85.00
GetMyObject	148.10	15.70	259.12	36.82	477.37	91.40	873.03	172.02	1139.78	309.60	1354.90	423.00

**Table 3:** Run native method in native.

From Java (ms)	100		200		400		600		800		1000	
	JNI	HPO	JNI	HPO	JNI	HPO	JNI	HPO	JNI	HPO	JNI	HPO
Sum	0.12	0.13	0.27	0.28	0.58	0.52	0.87	0.99	1.15	1.33	1.56	1.65
ArrayAdd	7.77	0.36	12.94	4.20	20.09	14.38	33.45	16.48	40.82	20.15	70.63	26.45
Strcat	6.16	0.40	17.02	4.82	33.84	16.28	54.84	20.26	98.93	24.19	127.34	29.77
GetMyObject	145.62	12.11	251.60	18.43	442.18	27.65	787.20	42.89	1054.20	51.34	1129.14	53.42

**Table 4:** Average elapsed time and invoking cost of native method.

From Java (ms)	From Java		In native		Invoking cost	
	JNI	HPO	JNI	HPO	JNI	HPO
Sum	2.06	2.45	1.40	1.04	0.66	1.41
ArrayAdd	78.33	43.76	61.66	23.28	16.67	20.48
Strcat	118.06	53.06	95.62	27.10	22.45	25.96
GetMyObject	1367.45	277.72	1233.09	79.40	134.36	198.32
